# Defining Translational Significance in Gerontology

**DOI:** 10.1093/geroni/igab046.761

**Published:** 2021-12-17

**Authors:** Steven Albert

**Affiliations:** University of Pittsburgh, Pittsburgh, Pennsylvania, United States

## Abstract

Innovation in Aging requires a statement from authors on translational significance. This requirement forces authors to consider the implications of their research for changing some component of aging. How does the research address a challenge posed by aging bodies, minds, relationships, or societies? The editorial board has developed criteria for assessing translational significance. Translational research must meet at least one of three criteria. It (i) must predict or explain a health or behavioral outcome, (ii) be advanced enough in deployment or development to assess these effects, and (iii) have a clear pathway to large-scale program delivery or change in clinical practice. The criteria rule out some kinds of submissions, such as scale development, single-case studies, or reviews of literature. We use these criteria to structure each article’s required translational significance statement. Rethinking translation may help focus research across the full set of GSA journals.

